# Morphologic design of nanostructures for enhanced antimicrobial activity

**DOI:** 10.1186/s12951-022-01733-x

**Published:** 2022-12-20

**Authors:** Fatma Al-Zahraa Sayed, Noura G. Eissa, Yidan Shen, David A. Hunstad, Karen L. Wooley, Mahmoud Elsabahy

**Affiliations:** 1 School of Biotechnology, Science Academy, Badr University in Cairo, Badr City, Cairo, 11829 Egypt; 2 Department of Pharmaceutics, Faculty of Pharmacy, Zagazig University, Zagazig, 44519 Egypt; 3 Departments of Chemistry, Materials Science and Engineering, and Chemical Engineering, Texas A&M University, College Station, TX 77842 USA; 4 Departments of Pediatrics and Molecular Microbiology, Washington University School of Medicine, St. Louis, MO 63110 USA; 5 Misr University for Science and Technology, 6th of October City, Cairo, 12566 Egypt

**Keywords:** Nanoparticles, Morphology, Antimicrobial, Shape design, Bacteria, Biomimicry

## Abstract

Despite significant progress in synthetic polymer chemistry and in control over tuning the structures and morphologies of nanoparticles, studies on morphologic design of nanomaterials for the purpose of optimizing antimicrobial activity have yielded mixed results. When designing antimicrobial materials, it is important to consider two distinctly different modes and mechanisms of activity—those that involve direct interactions with bacterial cells, and those that promote the entry of nanomaterials into infected host cells to gain access to intracellular pathogens. Antibacterial activity of nanoparticles may involve direct interactions with organisms and/or release of antibacterial cargo, and these activities depend on attractive interactions and contact areas between particles and bacterial or host cell surfaces, local curvature and dynamics of the particles, all of which are functions of nanoparticle shape. Bacteria may exist as spheres, rods, helices, or even in uncommon shapes (e.g., box- and star-shaped) and, furthermore, may transform into other morphologies along their lifespan. For bacteria that invade host cells, multivalent interactions are involved and are dependent upon bacterial size and shape. Therefore, mimicking bacterial shapes has been hypothesized to impact intracellular delivery of antimicrobial nanostructures. Indeed, designing complementarities between the shapes of microorganisms with nanoparticle platforms that are designed for antimicrobial delivery offers interesting new perspectives toward future nanomedicines. Some studies have reported improved antimicrobial activities with spherical shapes compared to non-spherical constructs, whereas other studies have reported higher activity for non-spherical structures (e.g., rod, discoid, cylinder, etc.). The shapes of nano- and microparticles have also been shown to impact their rates and extents of uptake by mammalian cells (macrophages, epithelial cells, and others). However, in most of these studies, nanoparticle morphology was not intentionally designed to mimic specific bacterial shape. Herein, the morphologic designs of nanoparticles that possess antimicrobial activities per se and those designed to deliver antimicrobial agent cargoes are reviewed. Furthermore, hypotheses beyond shape dependence and additional factors that help to explain apparent discrepancies among studies are highlighted.

**Graphical Abstract:**

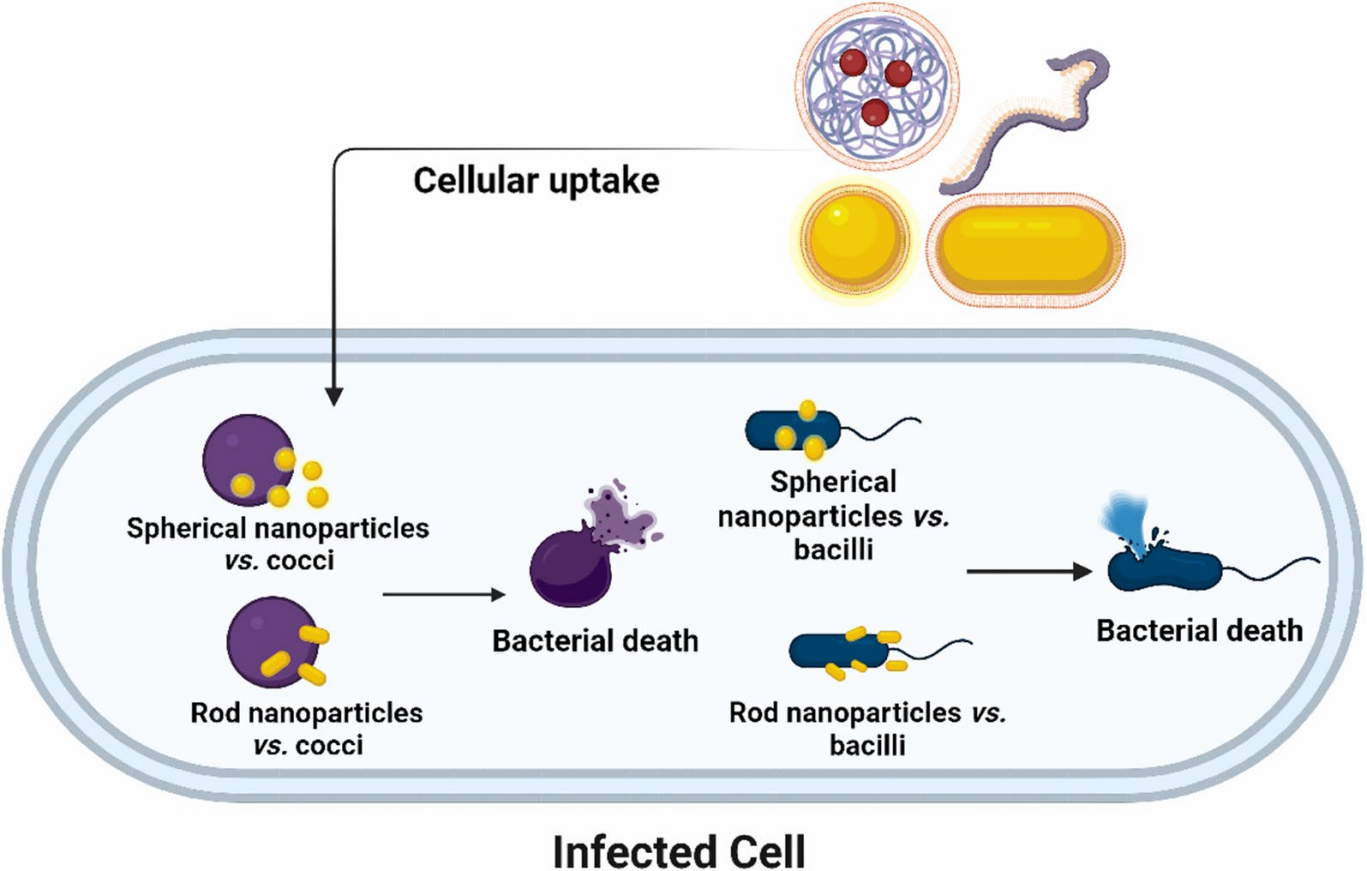

## Introduction

**Fig. 1:**
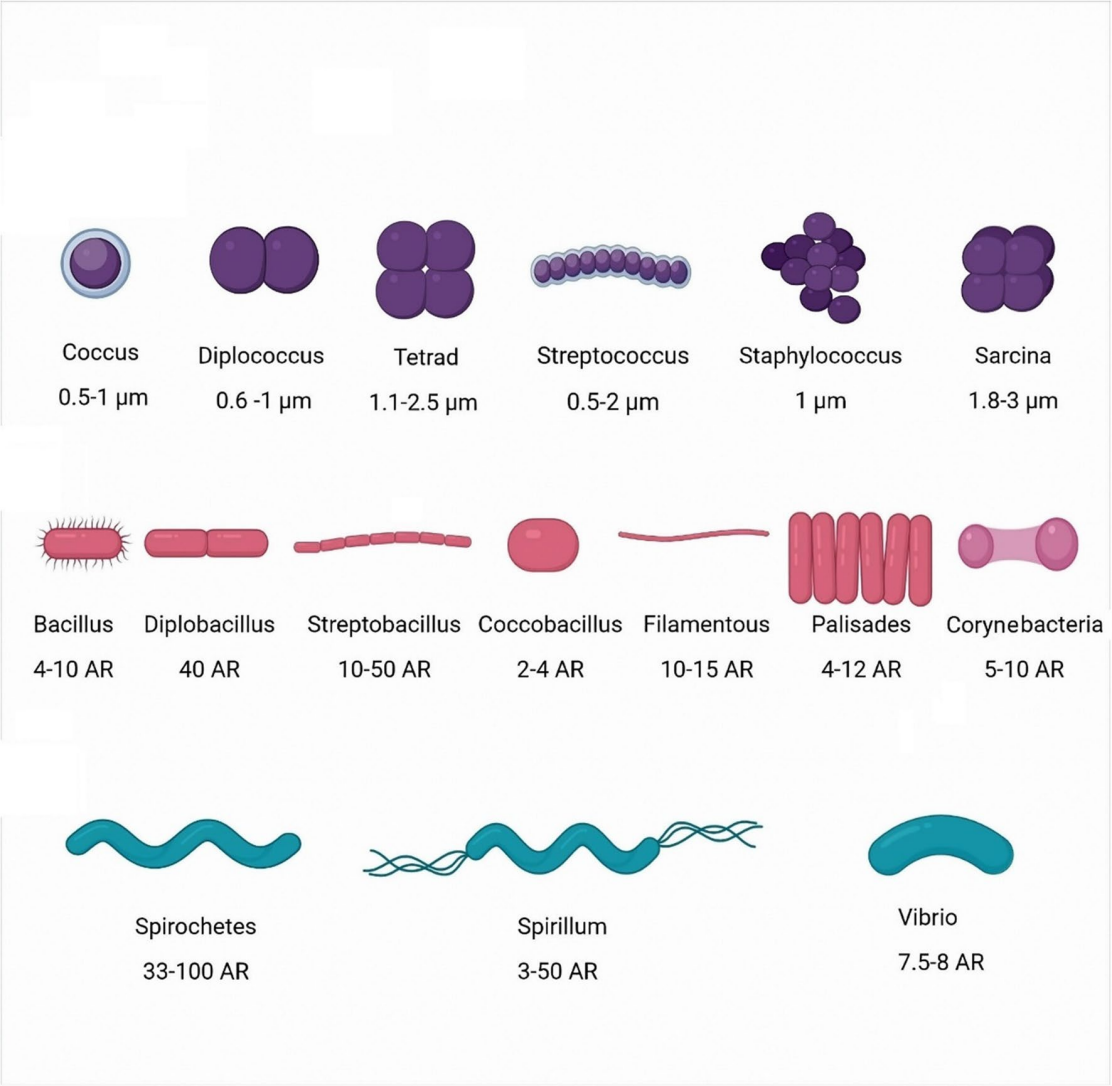
Common morphologies, arrangements and aspect ratios (AR) of cocci (Upper panel), bacilli (middle panel) and spiral (lower panel) bacteria. Figure created with Biorender.com

Bacterial shape is evolutionarily developed to support their existence, survival and proliferation under varying environments, interaction with other organisms, and formation of colonies and pathogenicity [1, 2]. Bacteria may exist as spheres, e.g., *N. gonorrhoeae,* rods, e.g., *E. coli*, helices, e.g., *H. pylori*, or even in uncommon shapes, such as box-shaped or star-shaped (Fig. 1) [3, 4]. Moreover, many bacteria may transform into other morphologies along their lifespan during adaptation to environmental cues. For instance, bacteria may develop long filamentous shapes (up to 10–50 times their original length) as a consequence of DNA damage, or upon exposure to stresses, such as antimicrobial agents. Filamentation by the rod-shaped *E. coli* within the mammalian urinary tract empowers bacteria to evade the immune system, as their long filamentous shape precludes engulfment by phagocytes [5–7]. Filamentous shape also facilitates transit between cells, perpetuating an intracellular niche that also shelters bacteria from phagocyte clearance and/or action by many antibiotic agents [8].

In 2017, the World Health Organization (WHO) announced that some strains of *A. baumannii*, a rod-shaped bacterium generally associated with hospital-acquired infections, have developed resistance to virtually all available antibiotics, including carbapenems, typically considered last-resort antibiotics [9]. This pathogen is just one of many that have rapidly assimilated multidrug resistance in recent years. For many reasons, including lack of market incentives, pharmaceutical companies have long prioritized development of therapeutics for chronic diseases and cancer over production of novel antibiotics. Existing antimicrobials are challenged by multiple factors including low solubility, bioavailability, bacterial resistance, and off-target toxicities [10]. The emergence of resistant bacteria, failure of existing antibiotics to conquer resistant strains, and limited development of new antibiotics, in addition to the sheltering of bacteria within host intracellular reservoirs noted above, all necessitate new measures to improve antibiotic delivery and antibacterial activities.

**Fig. 2:**
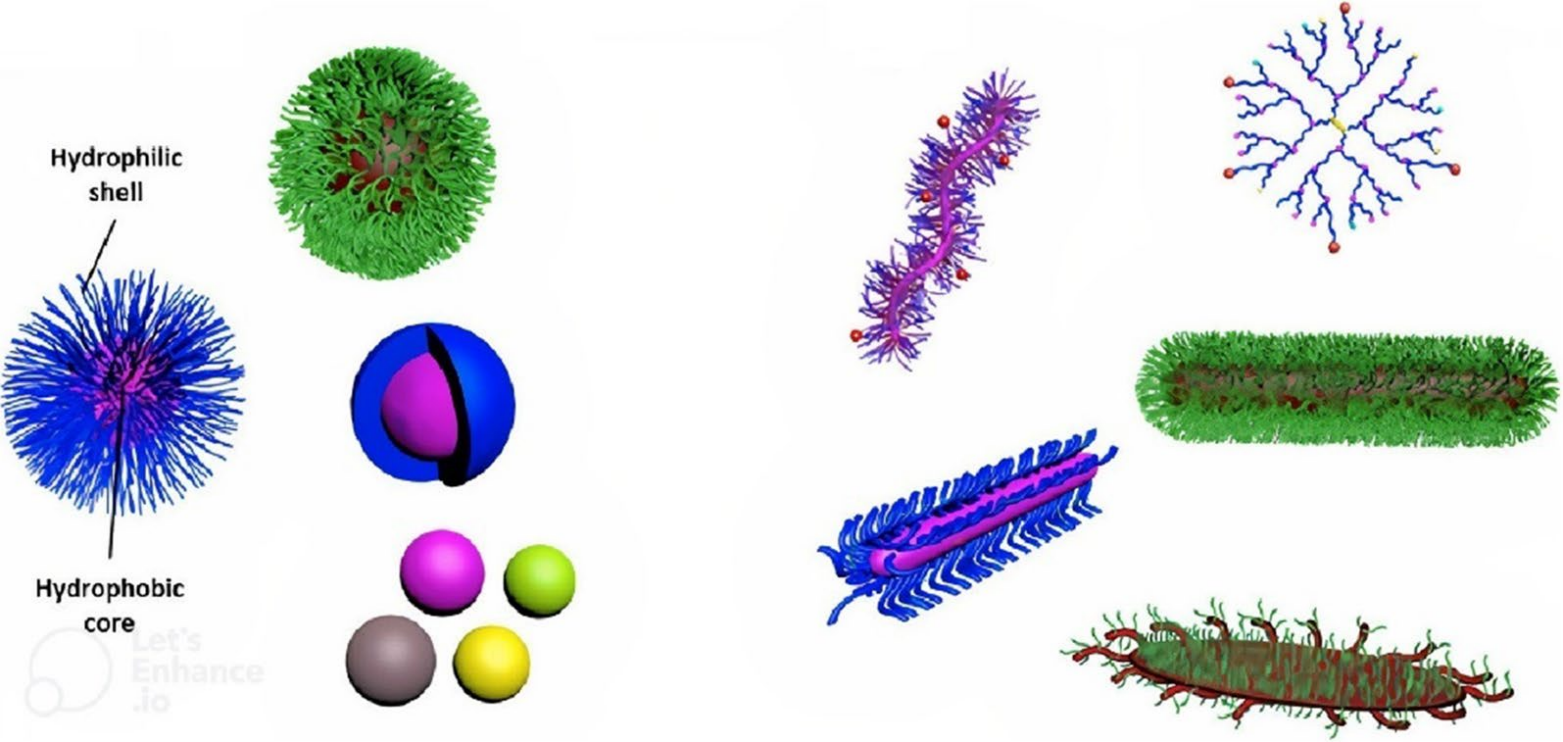
Nanoparticles of diverse morphologies and shapes that have been utilized for delivery of antibacterial cargoes: spherical nanoparticles of various types (e.g., polymeric, metallic) (left panel). Non-spherical nanoparticle shapes (e.g., cylindrical, platelet-like, unimolecular) and molecular topologies (e.g., hyperbranched structures) (right panel)

Nanoparticles can be used for delivery of existing antibiotic cargoes via multiple mechanisms of passive and/or active targeting [11, 12]. Whether acting by delivery of antibiotic cargo or possessing intrinsic antimicrobial activity, the effectiveness of nanoparticles can be enhanced by tailoring their physical and chemical characteristics (e.g., size, shape, surface chemistry, etc*.*), as reviewed previously by our group [13] and others [14–16]. In other words, shape-directed design of nanoparticles may not only enhance their affinity towards bacteria, but also increase their internalization into infected cells, particularly if combined with tailored surface chemistry. To mimic bacteria, nanoparticles can be synthesized and assembled into different shapes, such as spheres, rods, worm-like, discoidals, etc*.* (Fig. 2).

Extensive reviews have explored how nanoparticle shape may dictate cellular trafficking and internalization, as well as in vivo performance, for several biomedical applications [17, 18]. However, studies on the morphologic design of nanomaterials to mimic bacterial shape, and the effect of nanoparticle shape on antimicrobial efficiency, are limited. The current review highlights the main strategies that have been exploited to synthesize shape-tailored nanoparticles that possess superior antibacterial activities. Strategies and synthetic methods have mainly focused on incorporation of antimicrobial agents (e.g., silver) and on supramolecular assembly of polymeric building blocks into nanoparticles of defined shapes that are subsequently loaded with antimicrobial agents.

### Bacterial shape

The most familiar bacterial shapes are spheres, rods, and spirals (Fig. 1). Moreover, they exist in other uncommon structures, such as stars, pear-shaped, flask-shaped, and dendroid [19].

#### Spherical bacteria (Cocci)

The spherical shape is the simplest morphology adopted by bacteria [20, 21]. Coccoid bacteria divide along distinct planes, forming different multicellular arrangements, including *diplococci* (e.g.,* Neisseria gonorrhoeae* and *Moraxella catarrhalis*), chains (e.g.,* Streptococcus pyogenes* and *S. mutans*), tetrads (e.g.,* Micrococcus luteus*), grape-like clusters (e.g.,* Staphylococcus aureus*), and cuboidal forms (e.g.,* Sarcinae ventriculi*).

#### Rod-shaped bacteria (Bacilli)

Gram-positive bacteria adopting a rod shape include *Bacillus subtilis* [22], as well as spore-forming species such as *Bacillus cereus* and the anaerobe *Clostridium botulinum* [23, 24]*.* Gram-negative rods are widespread and include enteric bacteria (e.g., *Escherichia coli* and *Klebsiella* species) and environmental organisms (e.g., *Pseudomonas aeruginosa* and *Serratia marcescens*). Short rods, termed coccobacilli, are typified by the upper respiratory tract colonizer *Haemophilus influenzae* [25].

#### Helical (spiral) bacteria

Spiral bacteria may be classified as “spirochetes,” thin, long, spiral and flexible cells with internal flagella (e.g.,* Treponema, Spirochaeta, Leptospira and Borrelia spp*.); or “spirilla,” which are rigid structures with external flagella (*e.g., Helicobacter pylori*). Moreover, spiral bacteria include vibrio or comma-shaped bacteria (e.g.,* Vibrio cholerae* and *V. parahaemolyticus*) [20]. The spiral shape allows for accelerated motility of these bacteria in the gastrointestinal system and in media of high viscosity [26].

#### Bacterial morphology versus pathogenicity and resistance to antibacterial agents

The diverse morphologies displayed by bacteria play an essential role in their pathogenicity [2, 27]. For instance, helical shape and expression of flagella enable *H. pylori* to swim rapidly in viscous gastric mucin solutions, enhancing their ability to escape from the mucin gel [28]. Bacterial cell geometry is determined by certain cytoskeletal protein bundles that regulate cell membrane development and remodeling, such as, MreB, FtsZ and crescentin. For instance, *E. coli* can assume a spherical morphology upon depolymerization of MreB by small molecules that possess antibacterial activities (e.g., S-(3,4-dichlorobenzyl) isothiourea, also termed A22) [29, 30]. Bacteria may also adopt other morphologies along their lifespan. For example, *B. subtilis, S. aureus* and *Listeria monocytogenes* may shift to a cell wall-deficient state (“L-form”) upon treatment with cell-wall targeting antibiotics, such as β-lactams (e.g., penicillin and cephalosporin). This L-form switch allows bacteria to evade β-lactam action. Surprisingly, they can recover their walled state efficiently, enabling recurrent infections [31, 32]. Another example is *Caulobacter crescentus*, which changes its shape and aspect ratio upon exposure to sublethal doses of ribosome-targeting antibiotics [33].

### Generalized techniques to achieve tailored synthesis of shaped nanoparticles

**Fig. 3:**
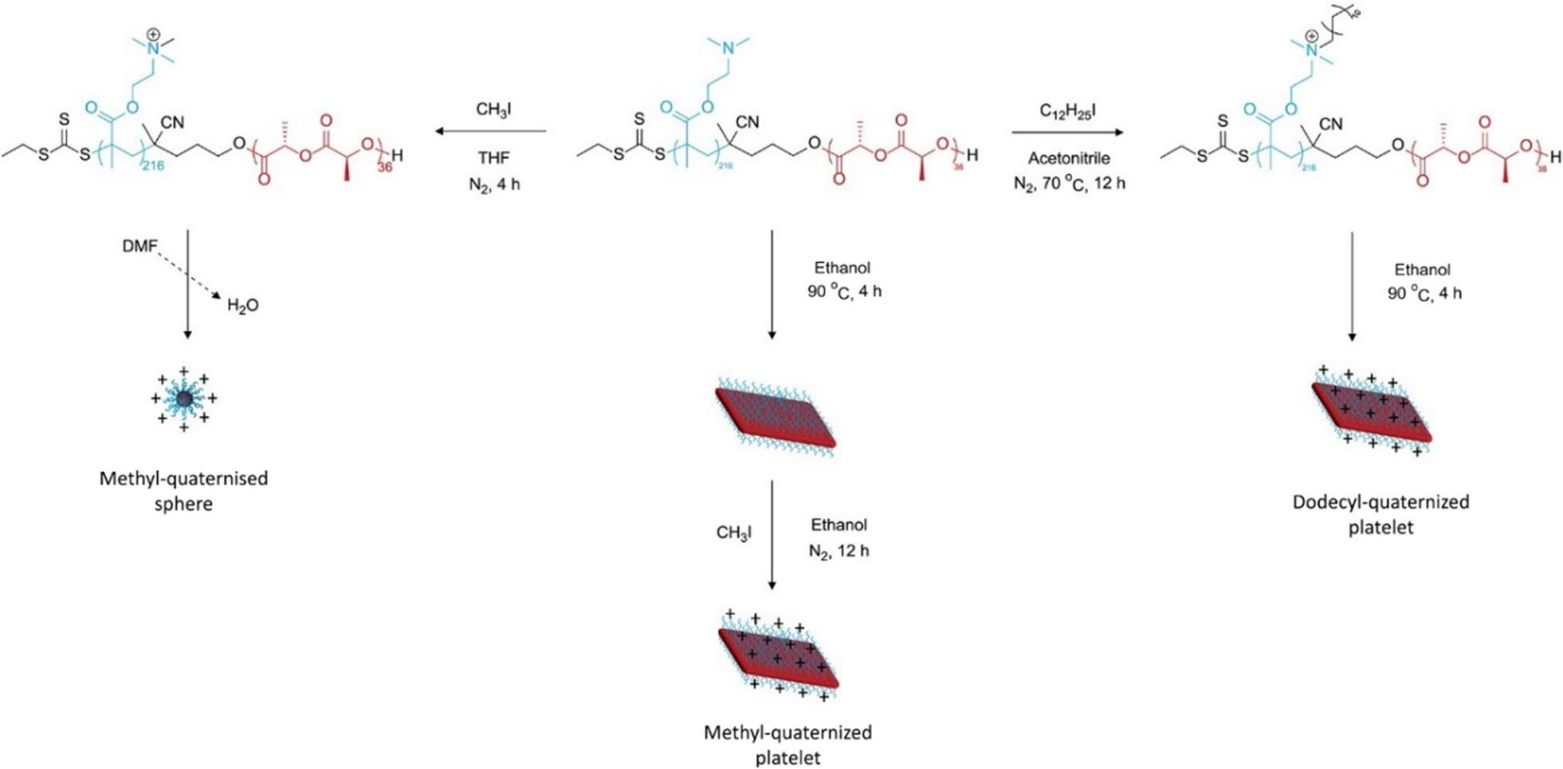
Preparation of quaternized PLLA_36_-*b*-PDMAEMA_216_ cationic spheres and crystalline platelets. Reproduced with permission from ref. [15]

**Fig. 4:**
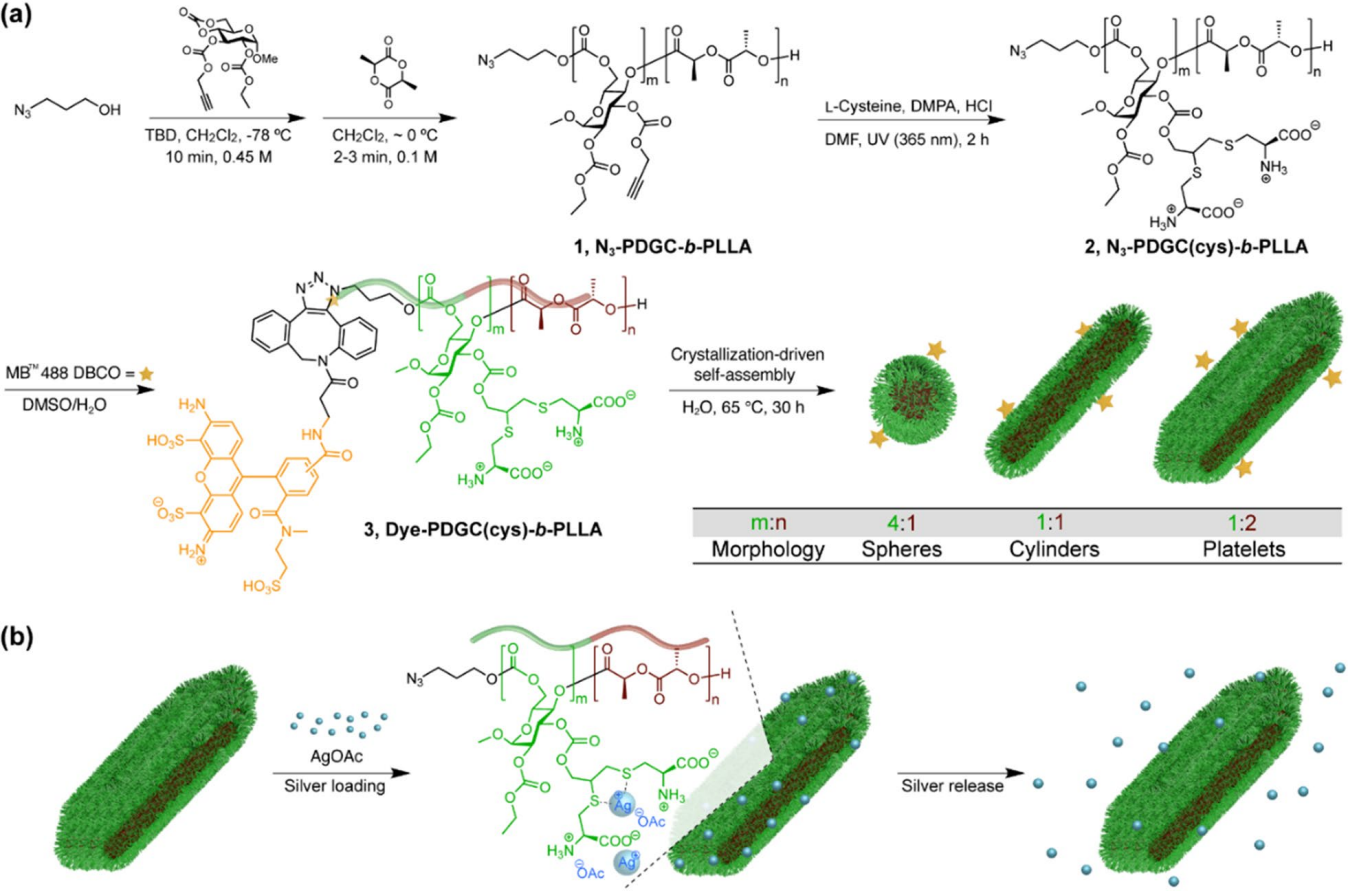
Synthetic schemes. **a** Synthesis of N_3_-PDGC-*b*-PLLA, by sequential ring-opening polymerizations of glucose-carbonate and l-lactide monomers from an azido-containing initiator, followed by post-polymerization modification via thiol-yne reaction with cysteine to prepare the zwitterionic N_3_-PDGC(cys)-*b*-PLLA, and chain-end modification of N_3_-PDGC(cys)-*b*-PLLA with dibenzocylcooctyne (DBCO)-functionalized fluorescent MB™ 488 to afford fluorescently-labeled PDGC(cys)-*b*-PLLA. **b** AgOAc loading into polymer nanostructures, through interactions with di-thioether and carboxylate groups, and release. Reprinted with permission from ref. [52]. Copyright (2022) American Chemical Society

Nanomaterials can be fabricated into versatile and complicated morphologies, including rods, worms, disks (circular, oblate, elliptical), bullets, barrels, etc*.* (Fig. 2) [34–36]. Nanoparticle pharmacokinetics (e.g., circulation lifetime), targeting, biodistribution, cellular uptake, and subsequent cellular trafficking are profoundly influenced by physicochemical characteristics of nanoparticles, such as morphology, size, and aspect ratio [37, 38]. Different methods have been utilized to synthesize nanoparticles of various morphologies for antimicrobial applications (Table 1, Figs. 3 and 4).

**Table 1:** Factors related to morphology and experimental/methodology setup that might influence antibacterial activities of nanostructures of diverse shapes

Type of nanoparticle	Shape	Size	Method of preparation	Bacteria	Antibacterial assessment method (concentration of nanoparticles)	Exposure time of bacteria to nanoparticles	References
Silver	Spheres Disks, Triangular plates	39 nm 51 nm 51 nm	Chemical reduction	*E. coli*, *S. aureus,* *P. aeruginosa*	Disk diffusion (0.01 mg/mL) and optical density growth curve (0.1–0.7 mg/mL)	24 h	[40]
Silver	Spheres Rods	40–60 nm 20–90 nm length × 20 nm width	Chemical reduction	*E. coli, S. aureus,* *B. subtilis,* *P. aeruginosa,* *K. pneumoniae*	Disk diffusion and (255–364 µg) minimum inhibitory concentration (184–358 µg/mL)	24 h	[68]
Silver	Spheres Rectangular Penta/hexagons	2–5 nm; 40–50 nm 40–65 nm 50–100 nm	Green synthesis (fungal)	*S. sonnei, E. coli,* *S. marcescens,* *S. aureus,* *P. aeruginosa*	Disk diffusion and colony count	24 h	[57]
Silver	Spheres Truncated octahedral	195 nm 194 nm	Chemical reduction	*E. coli,* *Enterococcus faecium*	Disk diffusion (100 µg/mL), optical density growth curve (50–1000 µg)	1–24 h	[70]
Silver	Nanospheres Nanocubes Nanowires	60 nm 55 nm (edge length) 60 nm diameter × 2–4 μm length	Chemical reduction (microwave-assisted)	*E. coli*	Optical density growth curve (12.5–50 µg/mL), minimum inhibitory concentration (3–100 µg/mL)	2–24 h	[42]
Silver	Plates Rods Hexagonal	N/A	Chemical reduction	*E. coli, S. aureus*	Disk diffusion (5.4 ppm), minimum inhibitory concentration (0–100 ppm), Colony count (10 ppm)	24 h	[41]
Silver-loaded polymer nanoparticles	Spheres Cylinders Platelets	11 nm 220 nm length × 20 nm diameter 620 nm length × 160 nm width	Crystallization-driven Self-Assembly	*E. coli* (uropathogenic strain UTI89 and laboratory strain MG1655)	Minimum inhibitory concentration (0.125–4 µg/mL Ag^+^)	24 h	[52]
Zinc oxide	Spheres Flower-like Rods	N/A	Solvothermal	*S. aureus, E. coli*	Colony count (20 ppm) –UV illumination	24 h	[44]
Zinc oxide	Spheres Hexagonal Cuboids	60–180 nm 65 nm 45 nm	Green synthesis (Aloe vera leaf extract)	*E. coli, B. subtilis,* *S. aureus*	Agar well diffusion, minimum inhibitory concentration, minimum bactericidal concentration	24 h	[56]
Tin oxide	Spheres Cauliflowers Flower petals	60–80 nm 96 nm 56 nm	Solvothermal	*E. coli*	Colony count – dark *vs.* visible-light *vs.* UV conditions	24 h	[43]
Quaternized poly(dimethyl-aminoethyl methacrylate)	Spheres Diamond-shaped platelets: Small Large	136 nm 600 nm 3700 nm	Crystallization-driven Self-Assembly	*E. coli, S. aureus,* *M. luteus,* *N. gonorrhoeae,* *P. aeruginosa,* *L. monocytogenes,* *B. subtilis*	Minimum inhibitory concentration (62.5–2500 µg/mL)	24–48 h	[15]

Chemical reduction and solvothermal methods have been commonly utilized for shape-directed synthesis of inorganic nanoparticles. Chemical reduction mainly includes an interaction of three main components: a precursor salt (e.g., AgNO_3_), a reducing agent (e.g*.*, _L_-ascorbic acid, sodium borohydride, sodium citrate, etc.), and a stabilizer (e.g., polyvinylpyrrolidone) [39–41]. Additionally, heating may be required during chemical reduction (e.g., via microwave-assisted method) [42]. Solvothermal methods has also been reported for the synthesis of metallic nanomaterials [e.g., zinc oxide (ZnO) and tin(IV) oxide (SnO_2_)] of varying morphologies, with or without the aid of surfactants [43, 44]. In these methods, solvents are an essential factor in controlling the shape of the synthesized nanoparticles, as solvent properties (e.g., polarity, viscosity) influence the solubility and dynamics of the precursors.

While the supramolecular assembly of amphiphilic block polymers has been studied extensively for many years and shown to allow for exquisite tuning of the overall nanoparticle size, shape and morphology, crystallization-driven self-assembly (CDSA) has emerged more recently as a technique to seed and grow polymer nanoparticles of ever more elaborate compositional and structural heterogeneities by controlled processes. Manners and Winnick pioneered CDSA as a powerful approach for the self-assembly of block copolymers in solution to afford formation of nanostructures of various morphologies [45–47] including those having well-defined dimensions with bar-coding [48] or scarf-like nanoobjects [49]. Importantly, O'Reilly and coworkers migrated the CDSA approach into aqueous solutions, which allows for study of the nanoparticle assemblies for biomedical applications directly, and demonstrated that the block length ratio can be tuned to allow formation of self-assemblies of different morphologies [15, 50, 51]*.* By such processes, intrinsically antibacterial cationic nanoparticles were produced by quaternization and CDSA of amphiphilic and cationic poly(l-lactide)_36_-*block*-poly(dimethylaminoethyl methacrylate)_216_ copolymers having a crystallizable PLLA block and a stabilizing PDMAEMA block. Quaternization of PLLA_36_-*b*-PDMAEMA_216_ by reaction with methyl iodide followed by CDSA in water afforded spherical micellar assemblies, whereas quaternization by reaction with lauryl iodide allowed for CDSA into platelets. To achieve platelet morphology with incorporation of methyl iodide, CDSA of (PLLA_36_-*b*-PDMAEMA_216_) was required prior to quaternization (Fig. 3) [15]. It was found that the platelet morphology in combination with surface chemistry imparted by the longer aliphatic chain lauryl-quaternized nanostructures resulted in the highest antibacterial activity. To enhance the biocompatibility, degradability, and sustainability of such nanostructured materials, Wooley and coworkers more recently synthesized amphiphilic block polymers for a similar CDSA morphological control study, but derived from naturally-sourced glucose and lactide with labile carbonate and ester linkages along the backbone. It was found that different hydrophilic-to-hydrophobic ratios of zwitterionic poly(d-glucose carbonate) and semicrystalline poly(l-lactide) segments afforded the formation of multifunctional polymer nanocarriers of tunable size and morphology (i.e., spheres, cylinders, and platelets) (Fig. 4) [52]. Ag was then loaded into polymer nanostructures, through interactions with di-thioether and carboxylate groups. In contrast to the O’Reilly system, these materials were not inherently cytotoxic, rather they were equipped with zwitterionic surface chemistries and designed to carry silver-based broadly antimicrobial cargoes for release [53–55].

Biological and sustainable methods utilizing plant extracts (e.g., aloe vera) [56] or non-pathogenic fungi (e.g.,* Trichoderma viride*) [57] have also been utilized to synthesize nanoparticles of different sizes and morphologies. These biological methods involve the use of various organisms (*e.g.,* bacteria, fungi and plants) that act as reducing and stabilizing agents [58]. Different parameters such as pH, temperature, reaction time, concentration of substrate and plant/fungal extract could be manipulated to develop nanoparticles of various sizes and morphologies.

### Effects of morphologic design of nanoparticles, specifically, to achieve enhanced antimicrobial activity

When designing antimicrobial materials, it is important to consider two distinctly different modes and mechanisms of activity—(i) those that involve direct interactions with bacterial cells, and (ii) those that promote entry of nanomaterials into infected host cells to gain access to intracellular pathogens. While spheres represent the nanoparticle morphology most commonly described in the literature, construction of non-spherical nanostructured assemblies of various morphologies can allow for mimicking cellular shapes (e.g., bacterial cells) for enhanced therapeutic effects and targeting of cargoes. In some cases it has been found that varying nanostructure morphology did not exhibit significant effect on microbial susceptibility or viability [59]. However, other studies by our group [52, 60] and others [61–63] have indeed demonstrated such effects, indicating that “one size or shape does not fit all.” The following sections review the shape-directed design of nanoparticles for antimicrobial delivery (Table 1).

#### (i) NP shapes and morphologies that involve direct interactions with bacterial cells

Various strategies and synthetic methods have been developed for controlling size and morphology of inorganic or organic/polymer nanoparticles to assess the effect of nanoparticle morphology on antimicrobial activity [64]. Similarity between the morphologies and aspect ratios of nanoparticles and bacteria may amplify opportunities for intimate contact and interaction [65, 66]. Many studies have involved inherently antimicrobial inorganic nanoparticles, e.g. those comprised of silver, while others involve the supramolecular assembly of amphiphilic building blocks to construct a scaffold that is then loaded with antimicrobial cargoes. For instance, a subclass of multi-molecularly assembled polymer nanomaterials with an extraordinary extended shape, termed filomicelles or filamentous micelles, possess high aspect ratio (1 µm length × 150 nm width) that might confer superior antibacterial activities upon loading with antimicrobial agents. Filomicelles may provide additional advantages in vivo as they have demonstrated a tenfold circulation lifetime compared to spherical analogs, due to a decreased rate of phagocytosis and clearance by the mononuclear phagocyte system [67].

Silver is a well-known potent and broad-spectrum antimicrobial that has been approved by the United States Food and Drug Administration (FDA) since the 1920s for wound management. Owing to their multiple modes of inhibitory actions, silver nanoparticles present a promising strategy for management of resistant microbial strains, alone or in combination with antibiotics (i.e., for a synergistic effect) [57]. Silver has been utilized in nanomedical technologies either as Ag^0^ nanoparticles that are able to shed Ag^+^ or as silver salts or silver complexes loaded as cargo within nanoparticle scaffolds.

**Fig. 5:**
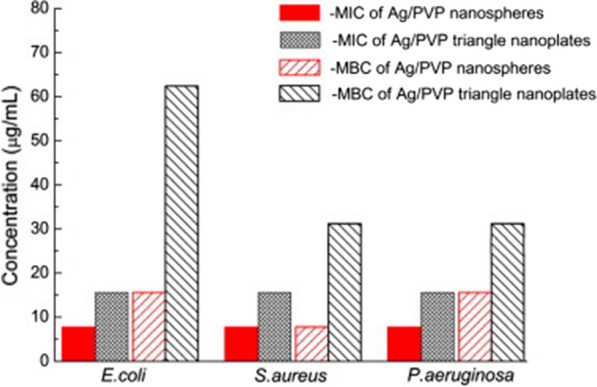
MIC and MBC histograms of Ag/PVP nanospheres and triangular nanoplates against E. coli, S. aureus and P. aeruginosa. Reprinted with permission from ref. [69]. Copyright (2022), with permission from Elsevier

**Fig. 6:**
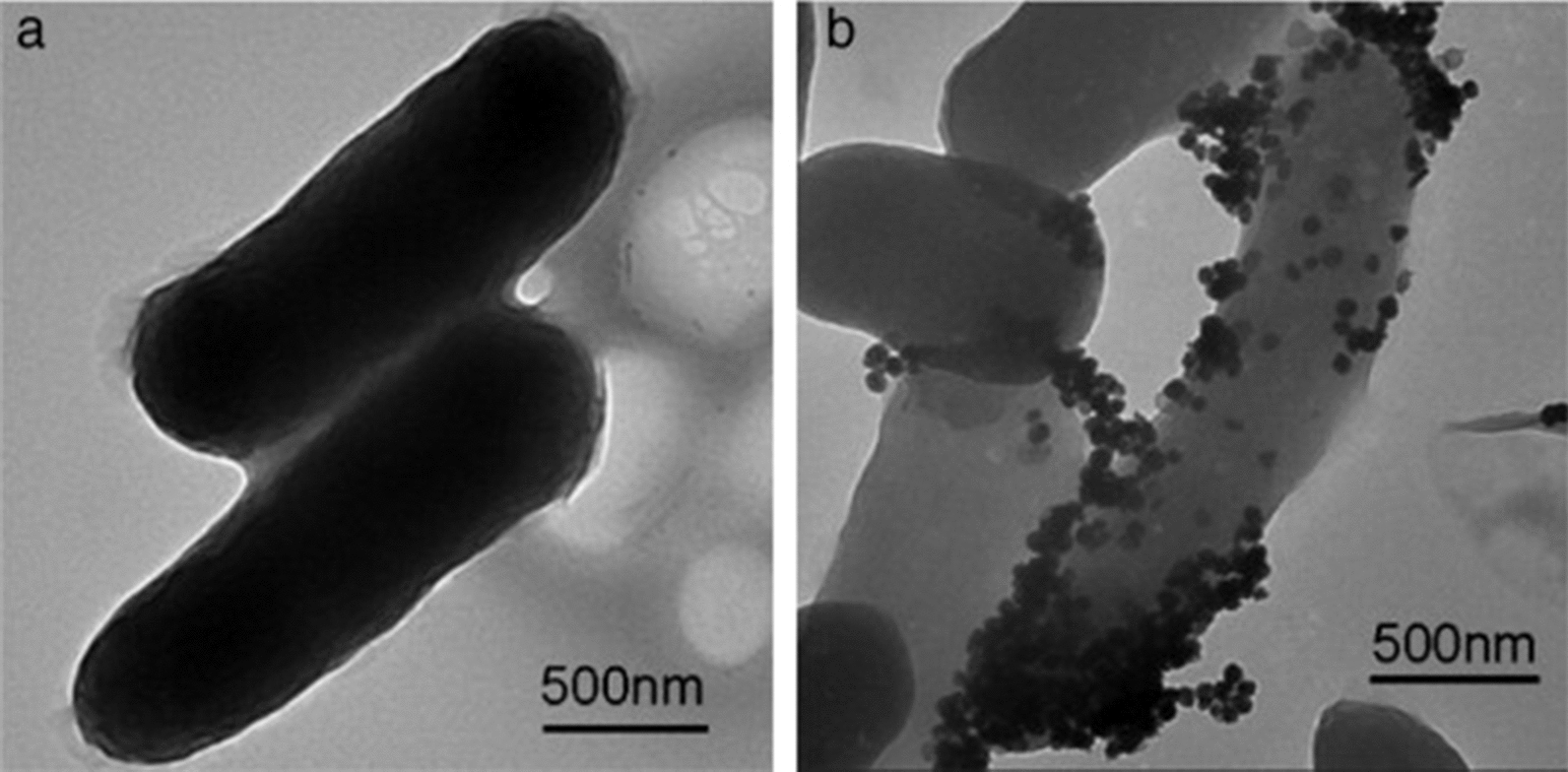
TEM images of **a** untreated E. coli and **b** E. coli treated with Ag/PVP triangular nanoplates. Reprinted with permission from ref. [69]. Copyright (2022), with permission from Elsevier

Interestingly, the size and morphology of silver nanoparticles were found to have considerable effects on their antimicrobial activities. For example, Cheon and coworkers synthesized Ag nanoparticles of different morphologies (i.e., spheres, disks and triangular plates), and examined their antimicrobial activities against *E. coli* [40]. The Ag nanoparticles of spherical, triangular plates and disks shapes displayed negative zeta potential of − 13.8 mV, − 28.6 mV and − 29.2 mV, respectively. Spherical Ag nanoparticles showed the most potent antimicrobial activity followed by disk-shaped nanoparticles, while triangular plates exerted the smallest zone of bacterial growth inhibition. This variability in the antimicrobial activities of Ag nanoparticles was attributed to differences in particle morphology and size, and consequently surface area. Spherical nanoparticles exhibited the highest surface area (1307 ± 5 cm^2^) and smallest size (38.5 nm), thereby allowing higher release rates of Ag^+^ ions and enhanced antimicrobial effects, compared to triangular plates that possessed the lowest surface area (1028 ± 35 cm^2^). In the Cheon study, surface areas were calculated based on the average particle sizes, which may not reflect differences in effective surface areas between nanoparticles of different shapes. In several other studies, surface areas were reported as areas *per* unit mass, which enhances comparability between various nanoparticles across the literature. In another study, spherical and rod-shaped Ag nanoparticles were synthesized, and their antibacterial activities against both Gram-positive and Gram-negative bacteria were evaluated. Both nanoparticle shapes exhibited significant antibacterial effects that varied according to the microbial species. Superior antibacterial activities were observed for spherical nanoparticles at lower minimal inhibitory concentration (MIC) values (Table 2). This effect was ascribed to greater distortion of the bacterial cell wall induced by the large surface area of spherical Ag nanoparticles that allowed closer contact with bacterial cells, compared to a rod morphology that resulted in looser contact with the bacterial cell wall [68]. The study also reported on the effect of the electrostatic interaction between the negatively charged Ag nanoparticles, stabilized with citrate, and the positively charged residues of the integral membrane proteins on the bacterial surface which results in alteration in the bacterial cell wall integrity and physicochemical properties resulting in the leakage of cytoplasmic contents and consequently cell death. A study by Gao and coworkers displayed excellent antimicrobial activities for both spherical and triangular Ag nanoparticles against *E. coli, S. aureus and P. aeruginosa*, with MIC values of ≤ 15.6 µg/mL (Fig. 5) [69]. Ag nanospheres possessed higher antibacterial activity than that of triangular nanoplates at the same tested concentration; this finding was attributed to the ability of nanospheres to achieve contact with bacteria more efficiently, thereby increasing the local concentration of released silver ions. The antimicrobial activity of triangular Ag nanoplates was attributed to the adsorption of the nanoparticles onto the bacterial cell membrane as illustrated by TEM (Fig. 6), thus enabling their penetration through bacterial cell membranes and the release of Ag^+^ which has a high affinity to react with phosphorous and sulfur proteins in the bacterial membrane. A study by Kumari and coworkers evaluated efficacy of Ag nanoparticles of different morphologies (spherical, rectangular, pentagonal, and hexagonal) and sizes (2–5 and 40–50 nm for spherical nanoparticles; 40–65 and 50–100 nm for rectangular and penta/hexagonal, respectively) [57]. The in vitro antibacterial effect of nanoparticles was evaluated alone or in combination with traditional antibiotics for management of multidrug-resistant pathogens. Among all shapes and sizes, small spherical nanoparticles most potently inhibited the tested pathogens (*E. coli, S. marcescens, P. aeruginosa,* and *Shigella sonnei*), followed by pentagonal and hexagonal nanoparticles. The potency of the spherical nanoparticles is attributed to their high surface area to volume ratio. Though triangular, pentagonal, and hexagonal particles were larger in size, they exhibited considerable antibacterial effect, which may be attributed to their sharp edges distorting the bacterial cell membrane. These nanoparticle preparations showed shape-independent synergistic antimicrobial activity when combined with antibiotics (i.e., streptomycin, kanamycin and tetracycline) against *S. marcescens*, a highly resistant species. Interestingly, shape- and size-dependent synergistic effects of Ag nanoparticles and antibiotics were noted only with those antibiotics against which the pathogen had gained resistance (i.e., ampicillin and penicillin).

**Table 2:** MIC values of spherical and rod-shaped Ag nanoparticles (AgNP-sp and AgNR, respectively) against different bacterial strains

Bacterial Strain	Strain no	AgNP-sp concentration in MIC value (µg/mL)	AgNR concentration in MIC value (µg/mL)
Gram-positive			
* S. aureus*	ATCC 25923	190	358
* B. subtilis*	AST5-2	195	350
Gram-negative			
* P. aeruginosa*	AL2-14B	188	348
* K. pneumoniae*	AWD5	184	320
* E. coli*	ATCC 25922	190	340

Reprinted with permission from ref. [68]. Copyright (2022) NATURE PORTFOLIO

**Fig. 7:**
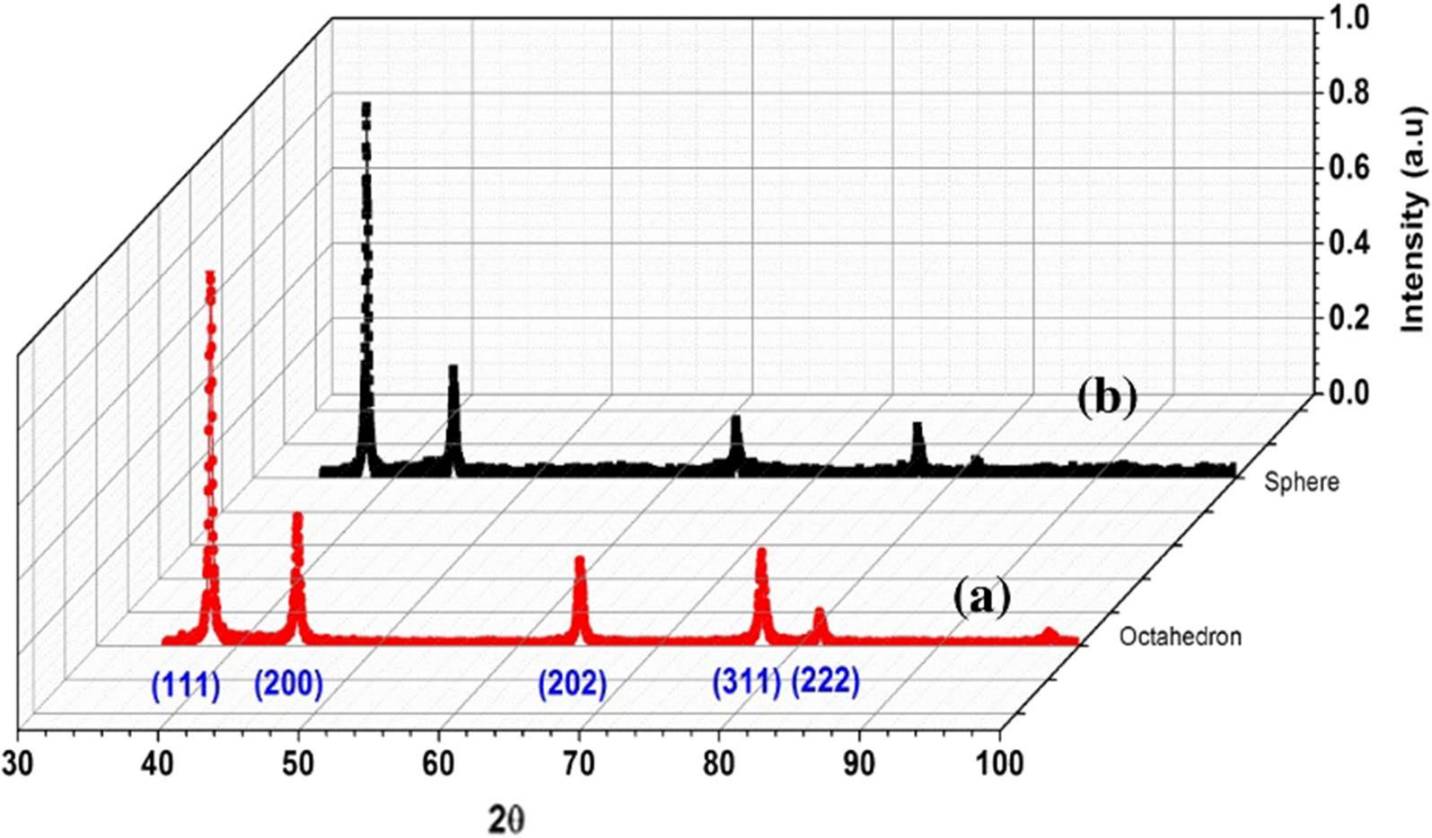
XRD pattern obtained from **a** octahedral and **b** spherical Ag nanoparticles. Reprinted with permission from ref. [70]. Copyright (2022), with permission from Elsevier

**Fig. 8:**
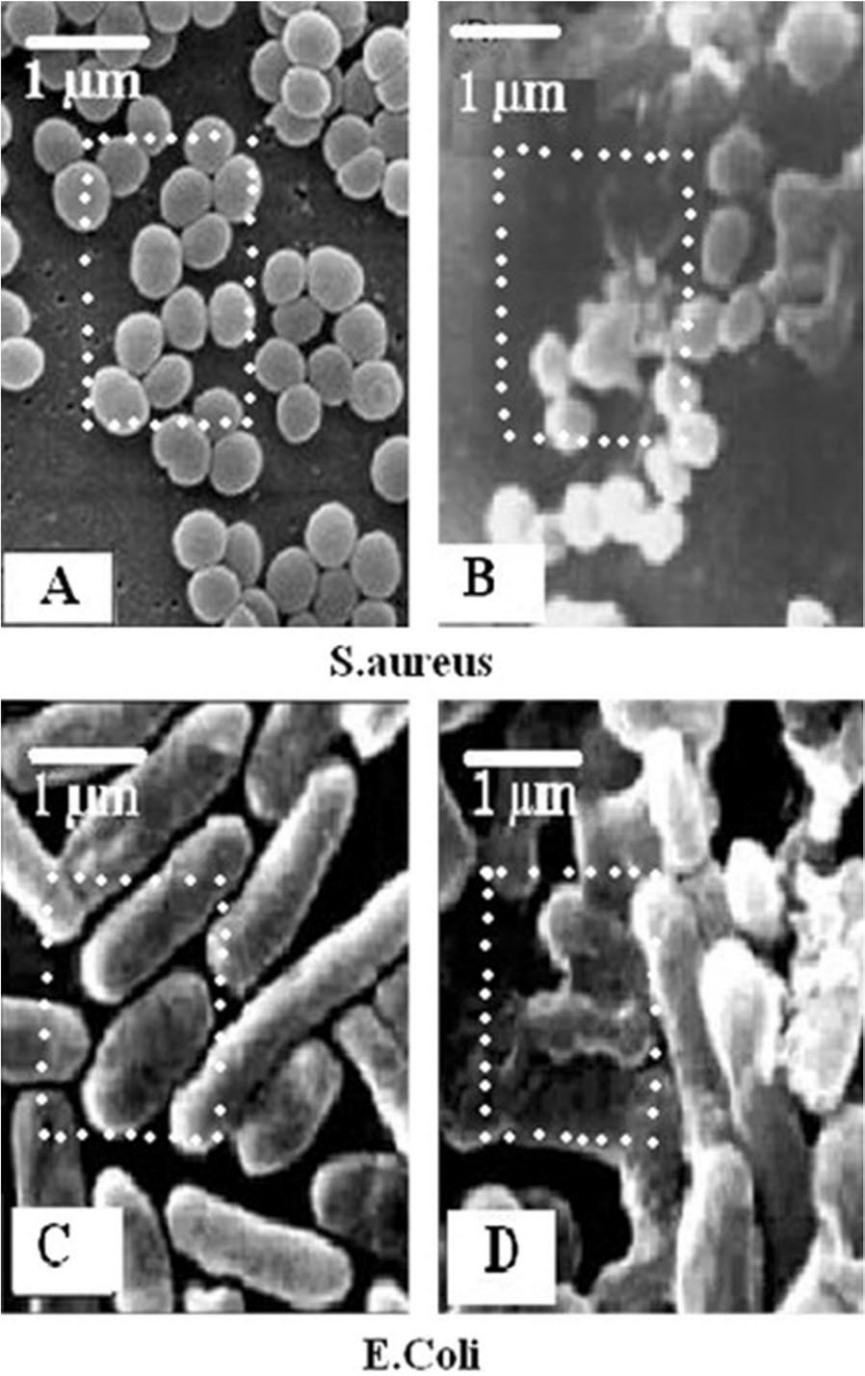
SEM images of *S. aureus* and *E. coli.* Normal cells (**A**, **C**) and cells (**B**, **D**) grown on LB agar containing nanosilver shape solutions (10 ppm). Reprinted with permission from ref. [41]. Copyright (2022), with permission from Elsevier

In some contrast to the above studies, Alshareef *et al*. reported higher bactericidal activity upon treatment of *E. coli* with truncated octahedral Ag nanoparticles as compared to treatment with their spherical counterparts [70]. The authors claimed that the differences in shape, active facets (i.e., flat faces on geometric shapes), and surface energies resulted in different antimicrobial efficacy. Specifically, octahedral nanoparticles exhibited higher surface area (1.32 m^2^/g) than spherical nanoparticles (1.26 m^2^/g). Furthermore, the geometric structure of truncated octahedral nanoparticles exhibited higher intensities of (111), (200), (202), (311) and (222) lattice planes, as confirmed by X-ray diffraction (XRD) data (utilizing Ag nanoparticles solutions) (Fig. 7), compared to spherical nanoparticles. Finally, the enhanced antibacterial activity of octahedral nanoparticles was also attributed to higher reactivity of atoms on the facets of higher surface energy, which led to rapid interactions with oxygen-containing groups of bacterial lipopolysaccharides. Another study examined the antibacterial activities of Ag nanoparticles of three different shapes (nanocubes, nanospheres, and nanowires) against *E. coli* [42]. Ag nanocubes exhibited the highest antibacterial activity and delayed the growth time of *E. coli* by 7 h at the lowest tested concentration (12.5 μg/mL), at which nanospheres and nanowires exerted no bacterial growth delay. Increasing the concentration of Ag nanoparticles to 50 μg/mL delayed the growth time by 12 h and 9 h for nanocubes and nanospheres, respectively. Nanowires, with the lowest surface area of the three constructs, showed the weakest antimicrobial activity, with only 6 h growth delay at the highest concentration (50 μg/mL) examined. On the contrary, nanocubes and nanospheres may allow closer effective contact with *E. coli* cells, thus inducing more cell membrane damage, and exhibiting a stronger antibacterial effect. Further, the authors proposed that the more highly reactive facets of nanocubes (compared to nanospheres) enabled nanocubes to locate on cell membranes more rapidly and induce cell membrane damage [42]. Hence, effective surface area still plays a critical role in dictating antimicrobial efficacy of nanomaterials, in addition to other contributing surface properties, i.e., active facets. Ardalan and coworkers synthesized Ag nanostructures with various morphologies (nanoplates, nanorods, and nanospheres) and investigated their antimicrobial activities, similarly finding that nanoplates exhibited the highest antibacterial activity against both *S. aureus* and *E. coli* [41]. This finding was attributed to the higher surface area of nanoplates (121 m^2^/g), compared to nanorods and hexagonal nanoparticles that possessed lower surface areas of 39 and 18 m^2^/g, respectively. SEM images revealed extensive damage of the bacterial strains treated with the Ag nanoparticles as compared to untreated cells (Fig. 8).

The application of Ag nanoparticles as broad-spectrum antimicrobials has expanded to other disciplines including the textile industry. To achieve higher quality and optimized production, and to reduce infections induced by human pathogens, industrial engineers are treating textiles with antimicrobial additives such as Ag nanoparticles [71, 72]. In this context, Nateghi and Hajimirzababa synthesized Ag nanoparticles of various morphologies (spheres, flat nanoplates, nanoprisms, polygonal, and hierarchical) and examined their antimicrobial activities on cotton fabrics against *E. coli* and *S. aureus* [73]. Non-spherical nanostructures (i.e., nanoprism, polygonal, and hierarchical) exhibited stronger growth inhibition effects as compared to spherical nanoparticles, with notably superior antimicrobial activity for hierarchical Ag nanoparticles (*ca.* 91% and 100% bacterial reduction percentages against *S. aureus* and *E. coli*, respectively). This was attributed to the high surface area of hierarchical nanoparticles (35 m^2^/g) that resulted in a more effective attachment of Ag nanoparticles to cotton fibers, as compared to the other morphologies of lower surface areas (18.2, 12.0, 17.6 and 14.8 m^2^/g for spherical, polygonal, prism, and disc nanoparticles, respectively). Spherical nanoparticles were found to be easily removed from the surface of cotton fibers more easily than the non-spherical morphologies due to less engagement within the coton fabrics.

Zinc oxide (ZnO) nanoparticles have been employed as efficient antimicrobial agents and as an economical alternative to metal nanoparticles (e.g., Ag nanoparticles) [74]. As with silver, ZnO nanoparticles can be synthesized in various morphologies that possess different antimicrobial efficacies. For instance, cuboidal ZnO nanoparticles exhibited more prominent antibacterial activities than spherical and hexagonal nanostructures [56]. Tin(IV) oxide (SnO_2_) is one of the metal oxides used in modern purification techniques and microbial photoinactivation. Talebian and Zavvare studied the antimicrobial activity of SnO_2_ nanostructures, synthesized using a solvothermal method in the presence or absence of surfactants, as a function of their structural and morphological characteristics [43]. Surfactant-mediated synthesis resulted in formation of spherical, cauliflower, and flower petal morphologies, while only spherical-like structures were observed with surfactant-free SnO_2_. Antibacterial activity of different nanoparticle morphologies varied under specific conditions (i.e., dark conditions, visible light, and UV). Under dark conditions or visible illumination, cauliflower and flower petal SnO_2_ nanoparticles were highly effective at initial bacterial inactivation in a relatively short time (˂ 10 min) compared to surfactant-mediated spherical SnO_2_ and spherical-like surfactant-free SnO_2_ nanostructures. On the contrary, under UV illumination, spherical-like surfactant-free SnO_2_ nanoparticles were most effective at initial bacterial inactivation, followed by surfactant-mediated spherical SnO_2_. These results provide evidence that multiple intertwined factors impact the antibacterial activities of synthesized nanostructures. In another study, the antibacterial activity of cupric oxide (CuO) nanospheres and nanosheets, was evaluated [75]. The results showed that nanosheets exhibited higher degrees of toxicity as compared to nanospheres and bulk CuO (i.e., at micro scale), attributed mainly to the morphology and size of nanosheets that enhanced physical and chemical mechanisms of toxicity. Irregular edges of nanosheets might contribute to perturbation of bacterial cell walls *via* physical interactions and puncturing. Additionally, nanosheets were found to have the greatest surface area and to possess higher electrochemical and surface catalytic reactivity.

Among many studies involving cationic polymers as antimicrobial agents, we highlight here just one study that probed effects of nanoparticle size and shape. Inam and co-authors synthesized quaternized PLLA_36_-*b*-PDMAEMA_216_ copolymers to afford formation of spherical (136 nm) and diamond-shaped platelets of two different sizes (i.e., small and large, 600 and 3700 nm, respectively), using a CDSA approach (Fig. 3) [15]. Small diamond-shaped platelets exhibited the highest antibacterial activity, compared to the large platelets and spherical structures. This observation was ascribed to the high charge density and compact size of small platelets that allowed tighter contact with the bacteria. Interestingly, higher antibacterial activity was observed for spherical nanoconstructs as compared to the large platelets, providing further evidence of interplay between size and morphology in dictating the antibacterial activity of synthesized nanostructures.

#### (ii) NP shapes and morphologies that allow nanomaterials to enter infected host cells to gain access to intracellular pathogens

**Fig. 9:**
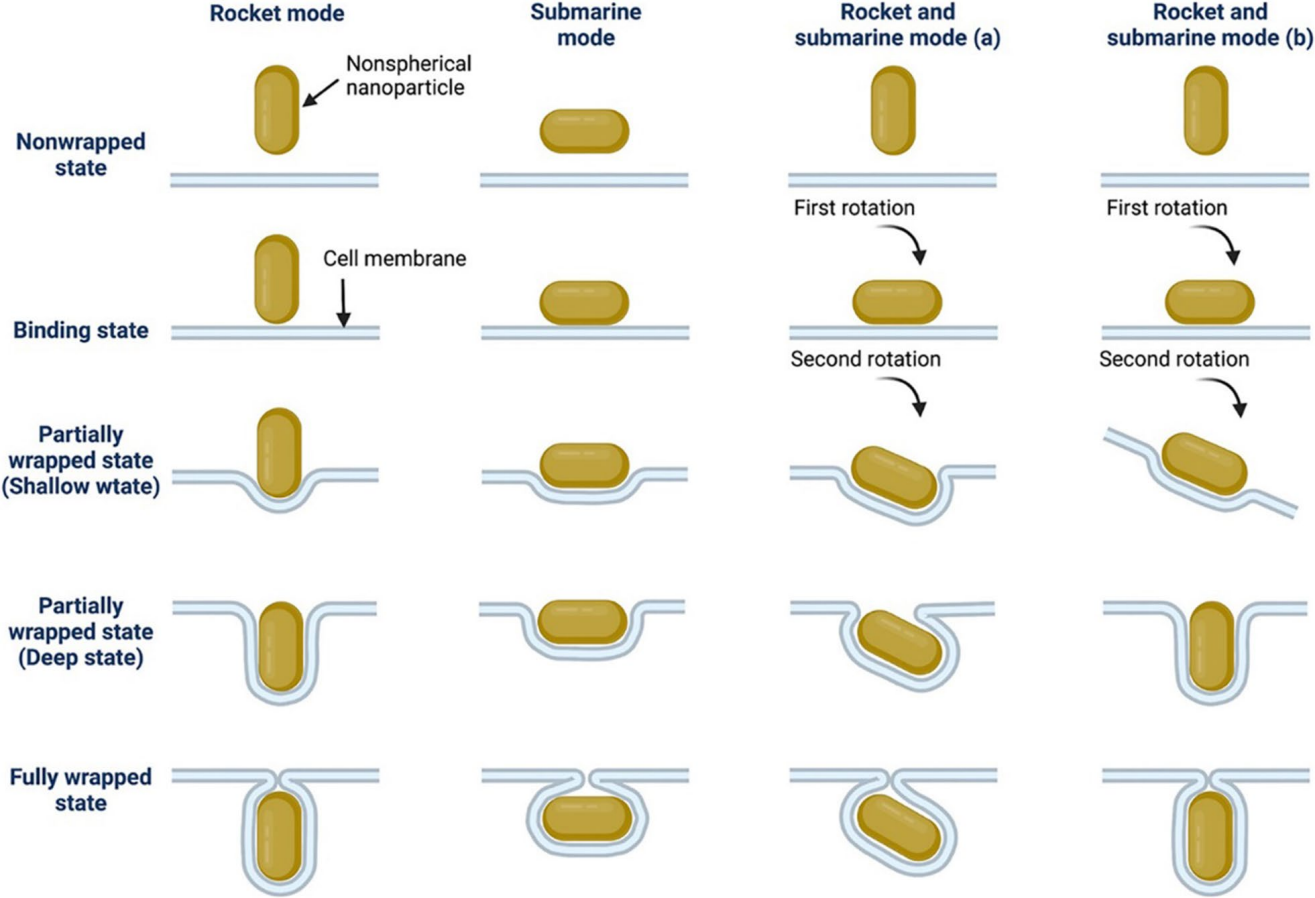
Illustration of the different entry modes of non-spherical nanoparticles according to numerical simulations. Reprinted from ref. [81], Copyright (2022), with permission from Elsevier

Physical translocation processes of nanoparticles of different morphologies (e.g., spherical, rods, discs, etc.) and volumes across lipid bilayers have been studied by exploiting computer simulations [76]. These results revealed that translocation of nanoparticles across lipid bilayers depends on the contact area between particle and membranes, local curvature of the particle and particle rotation, that are all reliant on nanoparticle shape [15, 76]. In parallel, research on the effect of nanoparticle shape on binding and uptake by live mammalian cells, and thus cargo delivery, has produced somewhat mixed results [77, 78]. Some studies have reported increased internalization for spherical shapes compared to non-spherical constructs [77, 79]. On the contrary, other studies have reported efficient cellular internalization of non-spherical structures (e.g., rod, discoid, cylinder, triangle sharp-shaped and quasi-ellipsoidal) compared to spherical particles [62, 80]. The shape and aspect ratio of nanoparticles have a crucial role in dictating the mechanism of cellular uptake (e.g., uptake *via* endocytosis or direct translocation) [81]. Cellular uptake can occur *via* phagocytosis, mainly by phagocytes of the immune system (e.g., neutrophils, macrophages), or pinocytosis *via* engulfment mechanisms that are classified according to the proteins and lipids involved (i.e., clathrin-mediated, caveolae-mediated, clathrin- and caveolae-independent endocytosis, and micropinocytosis). Nanoparticle shape contributes to the mechanism of interaction with cell membranes and their ultimate uptake by cells [82]. Endocytosis of a nanoparticle occurs *via* a dynamic process that includes several steps or stages (Fig. 9), which have been described as a nonwrapped state, a binding state, a partially wrapped state, and a completely wrapped state, resulting in the complete engulfment of the nanoparticle. For non-spherical nanoparticles (e.g., rod-shaped, disc-shaped, elliptical nanoparticles, etc.), the direction of approach may influence progression through those states, as illustrated in Fig. 9 [81]. Several reviews described the proposed mechanisms of antibacterial activities of various types of nanoparticles including metal nanoparticles [64, 83, 84]. These mechanisms include cell membrane penetration and damage, production of reactive oxygen species, cation release, biomolecule damages, and ATP depletion. As mentioned above, Wooley and coworkers recently synthesized biocompatible multifunctional polymer nanocarriers assembled from amphiphilic block copolymers, composed of zwitterionic poly(_D_-glucose carbonate) and semicrystalline polylactide segments, and loaded with silver cations (Fig. 4) [52]. CDSA with different hydrophilic-to-hydrophobic ratios afforded formation of nanostructures of diverse morphologies, i.e., spheres, cylinders, and platelets. The elongated cylindrical and platelet-like morphologies exhibited enhanced binding to uroepithelial cells, and cellular internalization was most evident with the platelet nanoparticles. These enhanced binding and internalization observations were attributed to the enlarged and elongated dimensions offered by non-spherical morphologies, which conferred improved and multivalent binding, favoring uroepithelial cell internalization despite their overall larger size. Interestingly, in addition to greater access to the intracellular environment of potentially infected host cells, Ag-loaded non-spherical nanoparticles (i.e., cylindrical and platelet-like) showed higher direct antibacterial potencies against two *E. coli* strains (uropathogenic strain UTI89 and laboratory strain MG1655) as compared to their spherical counterparts (Table 3).

**Table 3:** MICs (µg/mL Ag^+^) of silver acetate and silver-bearing polymer nanoparticles (Ag@spheres, Ag@cylinders, and Ag@platelets), against *E. coli* strains

	Formulation	*E. coli* Strains	
		UT189	MG1655
Average MIC (μg/mL)	AgOAc^a^	2.1 ± 0.9	2.2 ± 1.1
	Ag@Spheres	3.1 ± 0.4	3.1 ± 0.4
	Ag@Cylinders	1.7 ± 0.2	1.4 ± 0.3
	Ag@Platelets	2.1 ± 0.5	1.6 ± 0.2

Reprinted with permission from ref. [52]. Copyright (2022) American Chemical Society

^a^AgOAc MIC values from a prior study are shown for comparison

To actively target bacterial colonies within infected host cells, conjugation of bacterial adhesins (surface proteins that mediate bacterial binding to and internalization into host cells) provides a promising strategy for enhancing uptake of nanoparticles and their incorporated antimicrobial cargoes. In this context, Lin et al*.* synthesized amphiphilic block copolymers that were assembled into shell-crosslinked knedel-like spherical nanoparticles and were further conjugated with the terminal adhesive domain of the uropathogenic *E. coli* type 1 pilus adhesin, termed FimH [85]. This domain normally enables the pathogen to bind mannose moieties decorating the centers of the 16-nm uroplakin complexes found on the luminal surface of bladder epithelial cells. Fluorescent confocal microscopic examination demonstrated selective and dose-dependent binding of FimH_A_-decorated nanoparticles to bladder epithelial cells, whereas non-conjugated nanoparticles showed negligible cellular binding and uptake. These experiments demonstrate the concept of using a bacterium’s own strategy to achieve internalization of therapeutic nanoparticles into specific epithelial cell populations.

### Concluding remarks and perspectives

Recent decades have seen a wealth of progress in nanomaterial construction for a wide array of biomedical applications. Contemporary treatment of infectious diseases is challenged by burgeoning antibiotic resistance among prevalent global pathogens, as well as by delivery of antimicrobial agents to sites of infection at the needed concentrations and duration. Nanoparticle design offers potential future solutions to both of these obstacles, as antimicrobial or biocidal cargoes such as Ag^+^ can be released in very specific niches, and surface chemistries and conjugated moieties can help to optimize pharmacokinetics and pharmacodynamics of a nanostructure.

For infectious disease applications, the literature reports an array of studies that report seemingly disparate results regarding the optimal composition, size, morphology, and other features of therapeutic nanostructures for antimicrobial activity. However, these apparent discrepancies are likely attributable to many obvious and more subtle differences in particle construction, geometric features, and preparation methods used across the studies. These differences include those directly related to morphology (size, geometry, aspect ratio, availability of edges and faces), available surface area and methods of measurement, reactivity and functionality (surface charge and hydrophilicity, reactive surface chemistries, participation of surface moieties in multivalent interaction), and chemical features related to production (e.g., additives, excipients, surfactants, batch-to-batch variability). Moreover, variation in the methods used for testing antibacterial activity (microbial growth media composition, microbial density in the test wells, and bacterial species/strains and mammalian cell lines chosen) almost certainly introduce discrepancies that preclude simple conclusions about the “best” features of a nanoparticle for a particular application.

Small sized-nanoparticles, spherical and non-spherical, contribute to higher surface area allowing for tight contact with bacterial membranes, increased distortion of these membranes and higher release of therapeutic agents. Irregular edges of some non-spherical morphologies (e.g., nanosheets) might contribute to perturbation of bacterial cell walls via physical interactions and puncturing, and higher electrochemical and surface catalytic reactivity. Moreover, highly reactive facets of nanoparticles (e.g., nanocubes) demonstrated rapid interaction with cellular membranes and increased cellular membrane damage. It is worth mentioning that throughout the different studies that included nanomaterials of diverse morphologies, effective surface area played the major role in dictating their antimicrobial activities.

**Fig. 10:**
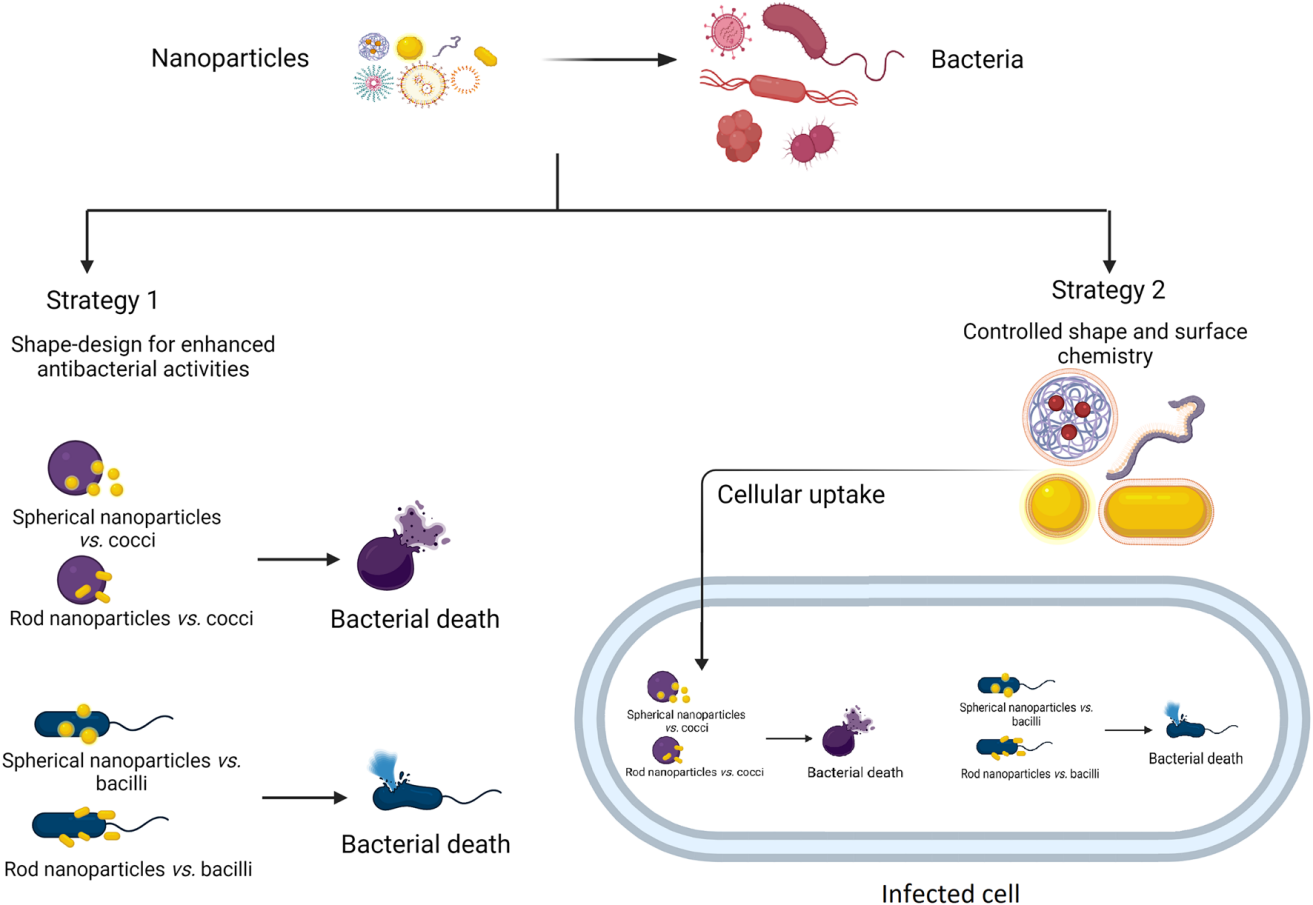
Shape-design strategies for enhanced antibacterial activities. One strategy depends on mimicking bacterial morphology to enhance bacterial attachment, disruption and/or uptake. Second strategy focuses on tuning shape and surface chemistry to maximize “selective” uptake into infected cells (e.g., bladder epithelial cells), followed by controlled interaction with the intracellular bacteria (e.g.,* E. coli*). Created with BioRender.com

Ultimately, the conceptual and practical “synthesis” of all of these features will be needed in order to develop therapeutically useful agents for human applications such as imaging and antimicrobial treatment. To date, for example, no studies have concurrently investigated the ability of synthesized nanoparticles to optimally bind and penetrate host cells (e.g., epithelial cells, macrophages, etc*.*) while also possessing the shape and other features that would maximize affinity/activity against bacteria encountered both within and outside those cells (Fig. 10). Future work aimed at understanding how nanoparticles of various shapes and morphologies interact with cell membranes, both mammalian and microbial, is crucial for the design of highly efficient antimicrobial nanostructures. It is also likely that each agent will have to be tailored specifically to the host niche and the pathogenic strategies used by a given pathogenic microbe. Continued progress in addressing these questions will help meet the global need for new weapons against multidrug-resistant human pathogens. A good start would be designing nanomaterials that possibly possess high effective surface area, active facets, expanded morphologies that allow intimate contact between nanoparticles and bacteria. Understanding the role of nanoparticle morphology will however rely on fixing the experimental conditions throughout the various studies, mainly the methods for manufacturing of the nanoparticles and for testing their antimicrobial efficacy.

## Data Availability

Not applicable.
